# PSYCHOLOGICAL RESILIENCE AMONG OLDER ADULTS WITH TRAUMATIC BRAIN INJURY: A CONCEPT ANALYSIS

**DOI:** 10.1093/geroni/igae098.3507

**Published:** 2024-12-31

**Authors:** Premgamon Kuntajak

**Affiliations:** University of Washington, Seattle, Washington, United States

## Abstract

**Aim:**

To examine the concept of psychological resilience among older adults with Traumatic Brain Injury (TBI).

**Background:**

Psychological resilience fosters adaptation after TBI, serving as a crucial indicator of recovery and success in rehabilitation. Despite being recognised for over decades, resilience remains a poorly defined concept within the realm of TBI. Thus, there is an urgent need for further exploration of psychological resilience within TBI to enhance the understanding and clarify the meaning of psychological resilience and its significance to older adults with TBI.

**Method:**

Walker and Avant’s modified method of concept analysis was used to guide this analysis. Literature from multiple scholarly databases was identified keywords: “Resilience,” “Psychological Adaptation,” “Hardiness,” and “Psychological Well-Being,” which were imputed using established inclusion criteria: full text, peer-reviewed, and printed in English from 2013-2023. Eligible articles were examined to determine the context of resilience and to comprehend and analyse its attributes, antecedents, and consequences. Outcomes: Antecedents of psychological resilience include exposure to adverse events and a social support system. Two principal attributes consisted of personal traits and the adaptation process. Psychological resilience facilitates improved physiological and psychological health outcomes, enhances recovery, and decreases complications among older adults with TBI.

**Conclusion:**

Research in TBI is transitioning from a deficit-oriented perspective to a strengths-based approach. A firm knowledge of psychological resilience from this analysis offers valuable insights that can guide nursing interventions, inform the application of theory, influence policymaking, and drive research possibilities aimed at improving health outcomes for older adults with TBI and their families.

